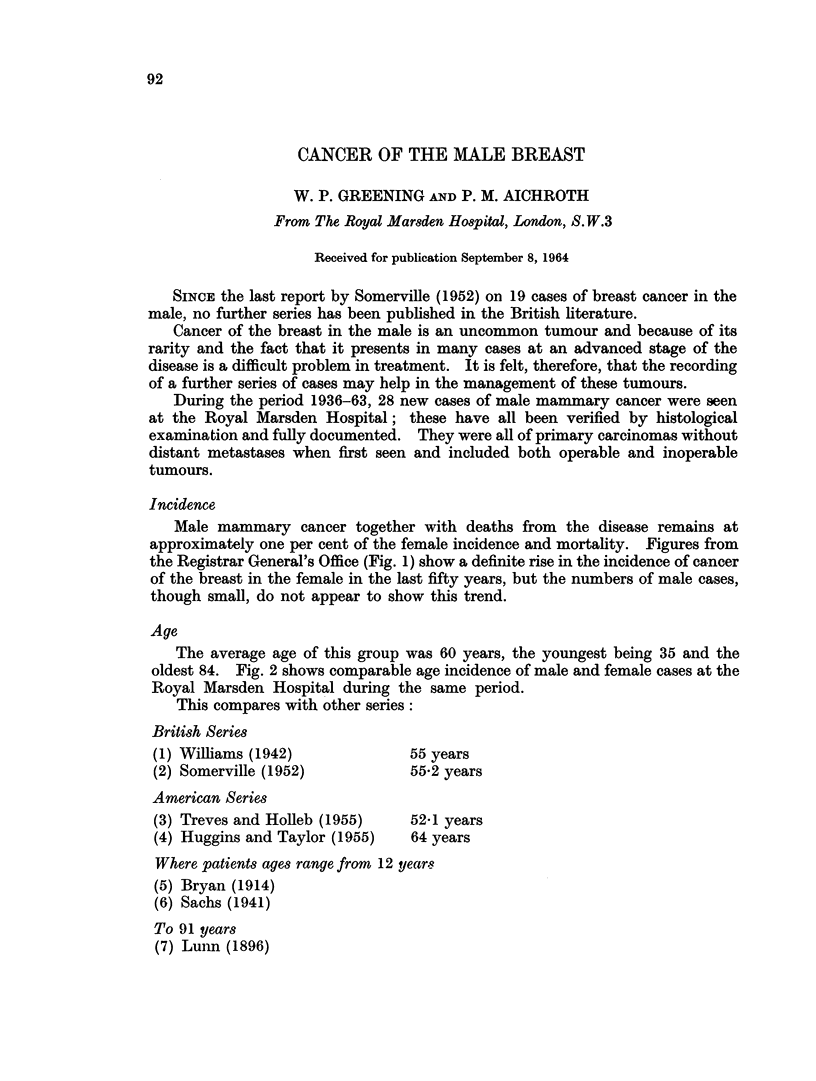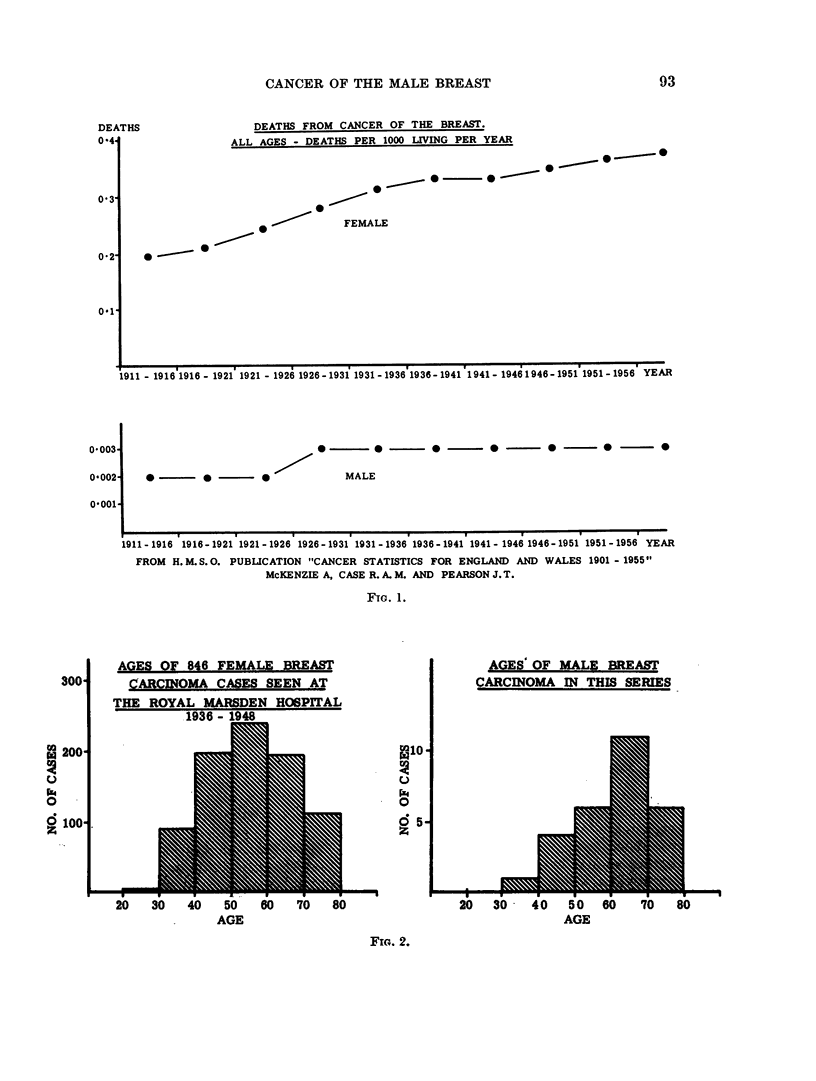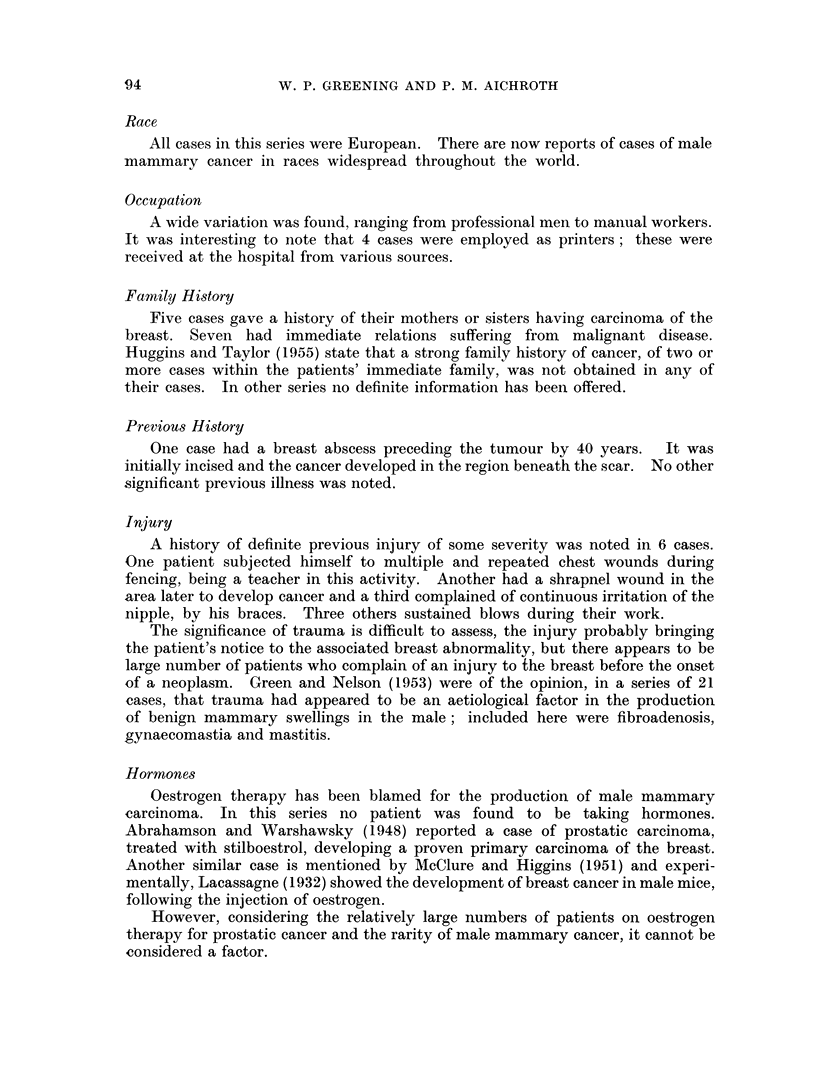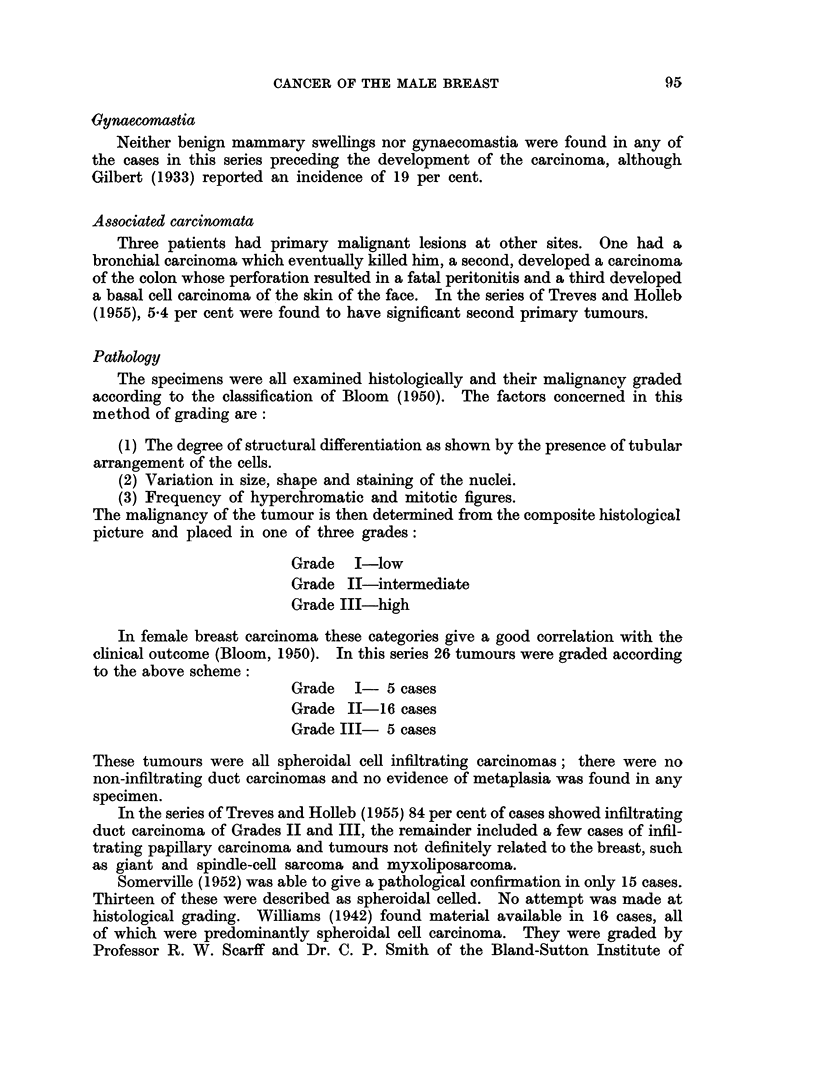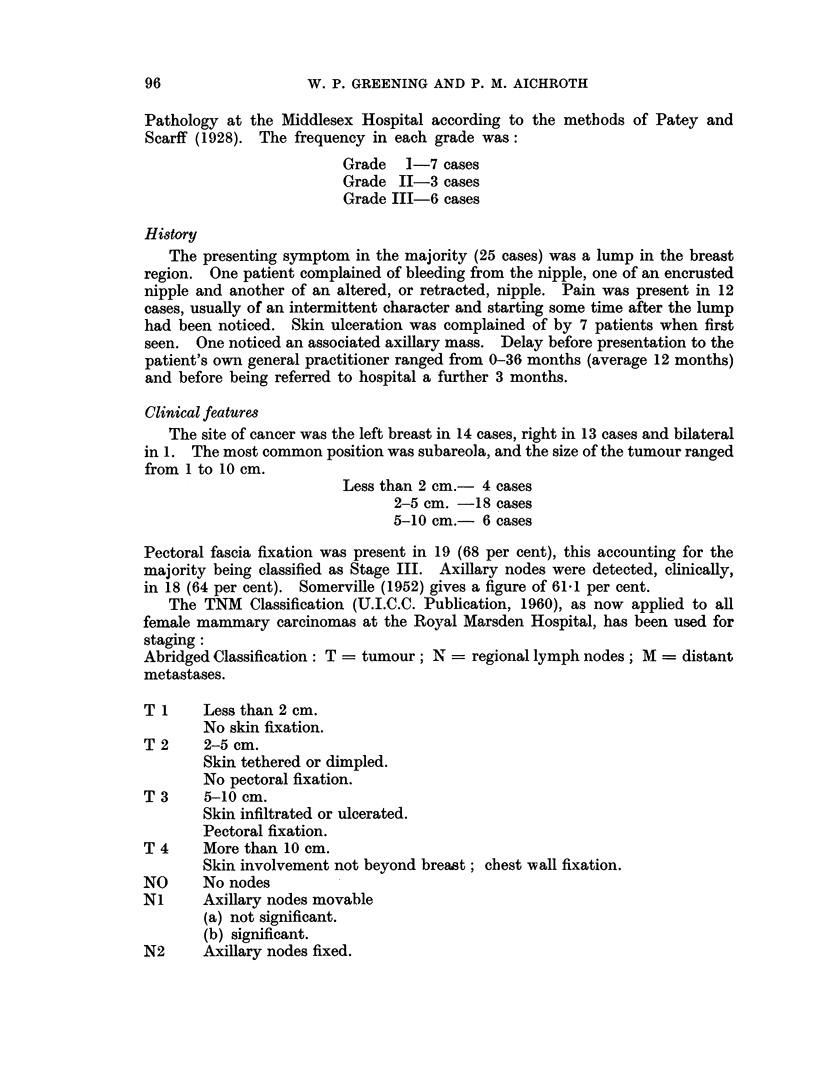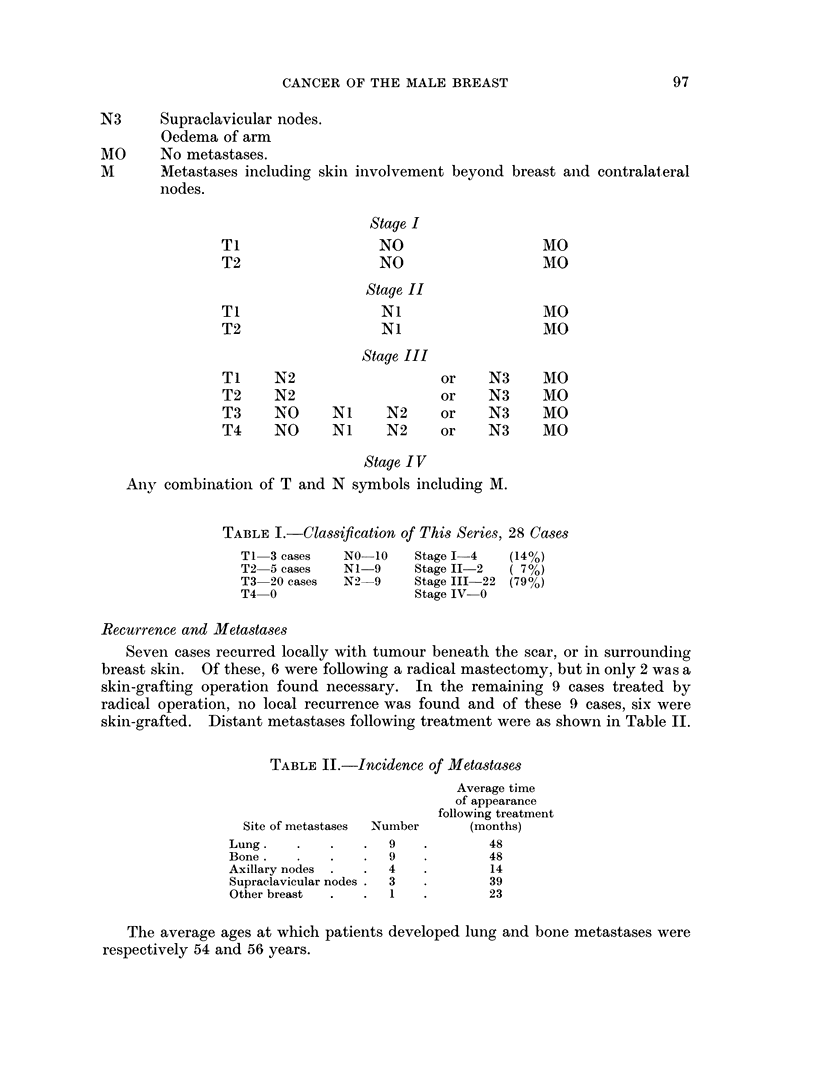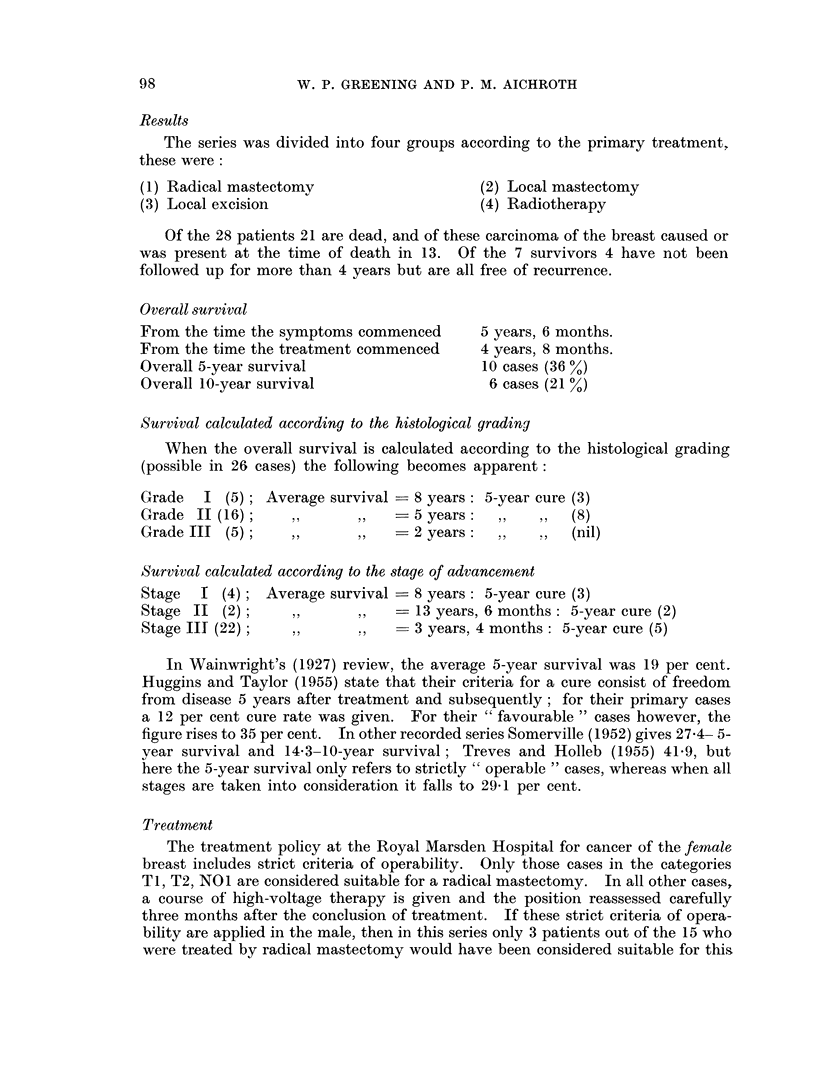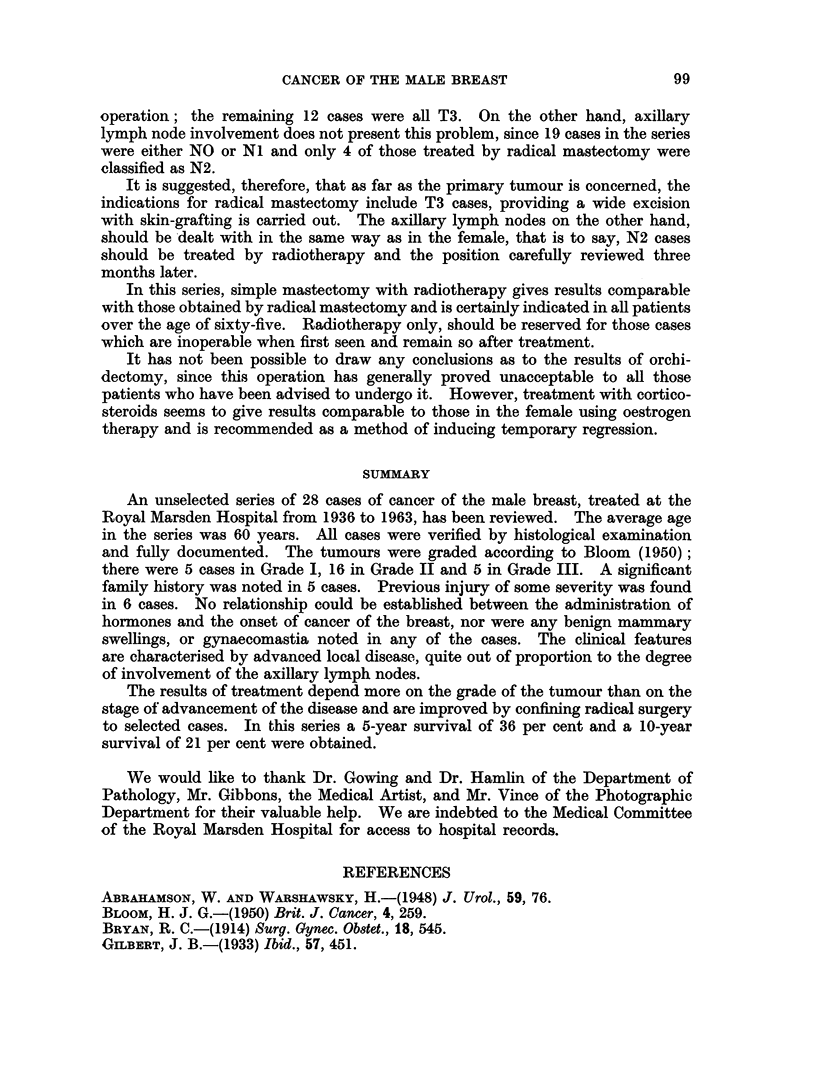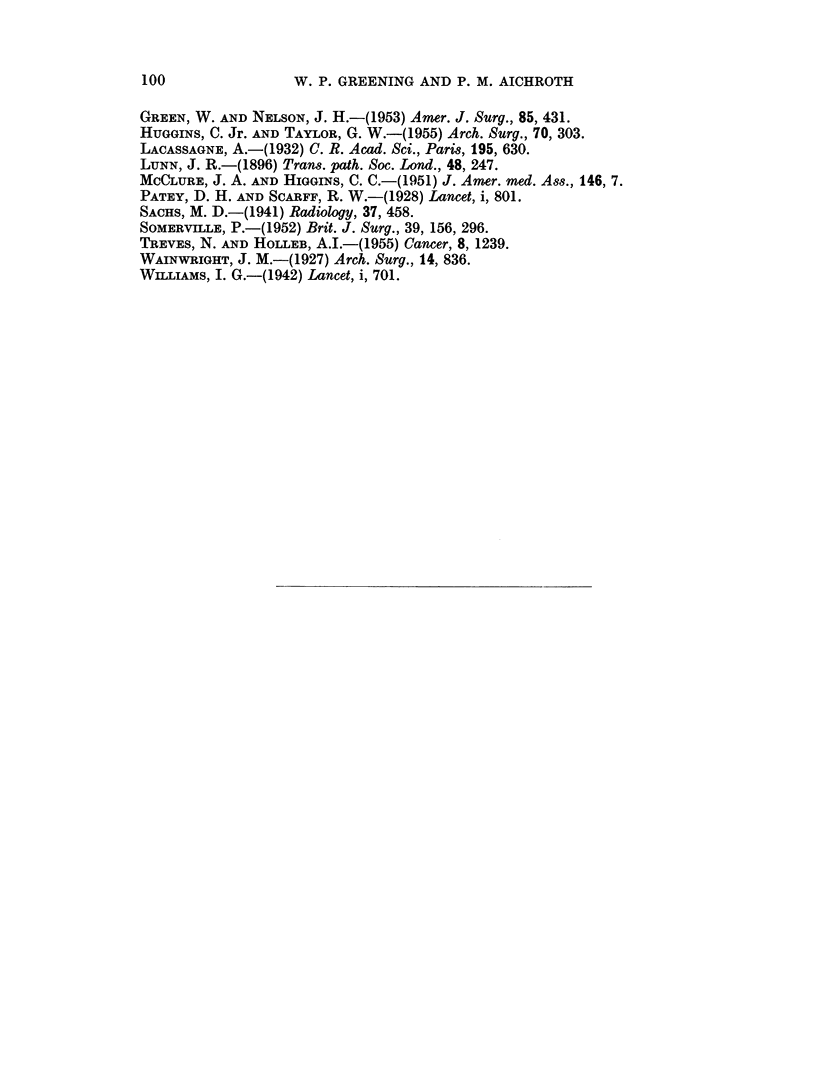# Cancer of the Male Breast

**DOI:** 10.1038/bjc.1965.9

**Published:** 1965-03

**Authors:** W. P. Greening, P. M. Aichroth


### CANCER OF THE MALE BREAST

W. P. GREENING AND P. M. AICHROTH

*From The Royal Marsden Hospital, London, S.W.3*

Received for publication September 8, 1964

SINCE the last report by Somerville (1952) on 19 cases of breast cancer in the male, no further series has been published in the British literature.

Cancer of the breast in the male is an uncommon tumour and because of its rarity and the fact that it presents in many cases at an advanced stage of the disease is a difficult problem in treatment. It is felt, therefore, that the recording of a further series of cases may help in the management of these tumours.

During the period 1936–63, 28 new cases of male mammary cancer were seen at the Royal Marsden Hospital; these have all been verified by histological examination and fully documented. They were all of primary carcinomas without distant metastases when first seen and included both operable and inoperable tumours.

### *Incidence*

Male mammary cancer together with deaths from the disease remains at approximately one per cent of the female incidence and mortality. Figures from the Registrar General's Office (Fig. 1) show a definite rise in the incidence of cancer of the breast in the female in the last fifty years, but the numbers of male cases, though small, do not appear to show this trend.

### *Age*

The average age of this group was 60 years, the youngest being 35 and the oldest 84. Fig. 2 shows comparable age incidence of male and female cases at the Royal Marsden Hospital during the same period.

This compares with other series:

*British Series*

| | |
|---|---|
| (1) Williams (1942) | 55 years |
| (2) Somerville (1952) | 55·2 years |

*American Series*

| | |
|---|---|
| (3) Treves and Holleb (1955) | 52·1 years |
| (4) Huggins and Taylor (1955) | 64 years |

*Where patients ages range from* 12 *years*

(5) Bryan (1914)
(6) Sachs (1941)

*To* 91 *years*

(7) Lunn (1896)

FROM H. M. S. O. PUBLICATION "CANCER STATISTICS FOR ENGLAND AND WALES 1901 - 1955"
McKENZIE A, CASE R. A. M. AND PEARSON J. T.

FIG. 1.

FIG. 2.

*Race*

All cases in this series were European. There are now reports of cases of male mammary cancer in races widespread throughout the world.

*Occupation*

A wide variation was found, ranging from professional men to manual workers. It was interesting to note that 4 cases were employed as printers ; these were received at the hospital from various sources.

*Family History*

Five cases gave a history of their mothers or sisters having carcinoma of the breast. Seven had immediate relations suffering from malignant disease. Huggins and Taylor (1955) state that a strong family history of cancer, of two or more cases within the patients' immediate family, was not obtained in any of their cases. In other series no definite information has been offered.

*Previous History*

One case had a breast abscess preceding the tumour by 40 years. It was initially incised and the cancer developed in the region beneath the scar. No other significant previous illness was noted.

*Injury*

A history of definite previous injury of some severity was noted in 6 cases. One patient subjected himself to multiple and repeated chest wounds during fencing, being a teacher in this activity. Another had a shrapnel wound in the area later to develop cancer and a third complained of continuous irritation of the nipple, by his braces. Three others sustained blows during their work.

The significance of trauma is difficult to assess, the injury probably bringing the patient's notice to the associated breast abnormality, but there appears to be large number of patients who complain of an injury to the breast before the onset of a neoplasm. Green and Nelson (1953) were of the opinion, in a series of 21 cases, that trauma had appeared to be an aetiological factor in the production of benign mammary swellings in the male ; included here were fibroadenosis, gynaecomastia and mastitis.

*Hormones*

Oestrogen therapy has been blamed for the production of male mammary carcinoma. In this series no patient was found to be taking hormones. Abrahamson and Warshawsky (1948) reported a case of prostatic carcinoma, treated with stilboestrol, developing a proven primary carcinoma of the breast. Another similar case is mentioned by McClure and Higgins (1951) and experimentally, Lacassagne (1932) showed the development of breast cancer in male mice, following the injection of oestrogen.

However, considering the relatively large numbers of patients on oestrogen therapy for prostatic cancer and the rarity of male mammary cancer, it cannot be considered a factor.

*Gynaecomastia*

Neither benign mammary swellings nor gynaecomastia were found in any of the cases in this series preceding the development of the carcinoma, although Gilbert (1933) reported an incidence of 19 per cent.

*Associated carcinomata*

Three patients had primary malignant lesions at other sites. One had a bronchial carcinoma which eventually killed him, a second, developed a carcinoma of the colon whose perforation resulted in a fatal peritonitis and a third developed a basal cell carcinoma of the skin of the face. In the series of Treves and Holleb (1955), 5·4 per cent were found to have significant second primary tumours.

*Pathology*

The specimens were all examined histologically and their malignancy graded according to the classification of Bloom (1950). The factors concerned in this method of grading are :

(1) The degree of structural differentiation as shown by the presence of tubular arrangement of the cells.
(2) Variation in size, shape and staining of the nuclei.
(3) Frequency of hyperchromatic and mitotic figures.

The malignancy of the tumour is then determined from the composite histological picture and placed in one of three grades :

Grade I—low
Grade II—intermediate
Grade III—high

In female breast carcinoma these categories give a good correlation with the clinical outcome (Bloom, 1950). In this series 26 tumours were graded according to the above scheme :

Grade I— 5 cases
Grade II—16 cases
Grade III— 5 cases

These tumours were all spheroidal cell infiltrating carcinomas ; there were no non-infiltrating duct carcinomas and no evidence of metaplasia was found in any specimen.

In the series of Treves and Holleb (1955) 84 per cent of cases showed infiltrating duct carcinoma of Grades II and III, the remainder included a few cases of infiltrating papillary carcinoma and tumours not definitely related to the breast, such as giant and spindle-cell sarcoma and myxoliposarcoma.

Somerville (1952) was able to give a pathological confirmation in only 15 cases. Thirteen of these were described as spheroidal celled. No attempt was made at histological grading. Williams (1942) found material available in 16 cases, all of which were predominantly spheroidal cell carcinoma. They were graded by Professor R. W. Scarff and Dr. C. P. Smith of the Bland-Sutton Institute of

Pathology at the Middlesex Hospital according to the methods of Patey and Scarff (1928). The frequency in each grade was:

Grade I—7 cases
Grade II—3 cases
Grade III—6 cases

*History*

The presenting symptom in the majority (25 cases) was a lump in the breast region. One patient complained of bleeding from the nipple, one of an encrusted nipple and another of an altered, or retracted, nipple. Pain was present in 12 cases, usually of an intermittent character and starting some time after the lump had been noticed. Skin ulceration was complained of by 7 patients when first seen. One noticed an associated axillary mass. Delay before presentation to the patient's own general practitioner ranged from 0–36 months (average 12 months) and before being referred to hospital a further 3 months.

*Clinical features*

The site of cancer was the left breast in 14 cases, right in 13 cases and bilateral in 1. The most common position was subareola, and the size of the tumour ranged from 1 to 10 cm.

Less than 2 cm.— 4 cases
2–5 cm. —18 cases
5–10 cm.— 6 cases

Pectoral fascia fixation was present in 19 (68 per cent), this accounting for the majority being classified as Stage III. Axillary nodes were detected, clinically, in 18 (64 per cent). Somerville (1952) gives a figure of 61·1 per cent.

The TNM Classification (U.I.C.C. Publication, 1960), as now applied to all female mammary carcinomas at the Royal Marsden Hospital, has been used for staging:

Abridged Classification: T = tumour; N = regional lymph nodes; M = distant metastases.

T 1 Less than 2 cm.
No skin fixation.

T 2 2–5 cm.
Skin tethered or dimpled.
No pectoral fixation.

T 3 5–10 cm.
Skin infiltrated or ulcerated.
Pectoral fixation.

T 4 More than 10 cm.
Skin involvement not beyond breast; chest wall fixation.

NO No nodes

N1 Axillary nodes movable
(a) not significant.
(b) significant.

N2 Axillary nodes fixed.

| | |
|---|---|
| N3 | Supraclavicular nodes. |
| | Oedema of arm |
| MO | No metastases. |
| M | Metastases including skin involvement beyond breast and contralateral nodes. |

*Stage I*

| | | |
|---|---|---|
| T1 | NO | MO |
| T2 | NO | MO |

*Stage II*

| | | |
|---|---|---|
| T1 | N1 | MO |
| T2 | N1 | MO |

*Stage III*

| | | | | | | |
|---|---|---|---|---|---|---|
| T1 | N2 | | | or | N3 | MO |
| T2 | N2 | | | or | N3 | MO |
| T3 | NO | N1 | N2 | or | N3 | MO |
| T4 | NO | N1 | N2 | or | N3 | MO |

*Stage IV*

Any combination of T and N symbols including M.

TABLE I.—*Classification of This Series*, 28 *Cases*

| | | | |
|---|---|---|---|
| T1—3 cases | N0—10 | Stage I—4 | (14%) |
| T2—5 cases | N1—9 | Stage II—2 | ( 7%) |
| T3—20 cases | N2—9 | Stage III—22 | (79%) |
| T4—0 | | Stage IV—0 | |

*Recurrence and Metastases*

Seven cases recurred locally with tumour beneath the scar, or in surrounding breast skin. Of these, 6 were following a radical mastectomy, but in only 2 was a skin-grafting operation found necessary. In the remaining 9 cases treated by radical operation, no local recurrence was found and of these 9 cases, six were skin-grafted. Distant metastases following treatment were as shown in Table II.

TABLE II.—*Incidence of Metastases*

| Site of metastases | Number | Average time of appearance following treatment (months) |
|---|---|---|
| Lung . . . . | 9 . | 48 |
| Bone . . . . | 9 . | 48 |
| Axillary nodes . . | 4 . | 14 |
| Supraclavicular nodes . | 3 . | 39 |
| Other breast . . | 1 . | 23 |

The average ages at which patients developed lung and bone metastases were respectively 54 and 56 years.

*Results*

The series was divided into four groups according to the primary treatment, these were :

| | |
|---|---|
| (1) Radical mastectomy | (2) Local mastectomy |
| (3) Local excision | (4) Radiotherapy |

Of the 28 patients 21 are dead, and of these carcinoma of the breast caused or was present at the time of death in 13. Of the 7 survivors 4 have not been followed up for more than 4 years but are all free of recurrence.

*Overall survival*

| | |
|---|---|
| From the time the symptoms commenced | 5 years, 6 months. |
| From the time the treatment commenced | 4 years, 8 months. |
| Overall 5-year survival | 10 cases (36 %) |
| Overall 10-year survival | 6 cases (21 %) |

*Survival calculated according to the histological grading*

When the overall survival is calculated according to the histological grading (possible in 26 cases) the following becomes apparent :

| | | | | |
|---|---|---|---|---|
| Grade I (5) ; | Average survival | = 8 years : | 5-year cure | (3) |
| Grade II (16) ; | ,, ,, | = 5 years : | ,, ,, | (8) |
| Grade III (5) ; | ,, ,, | = 2 years : | ,, ,, | (nil) |

*Survival calculated according to the stage of advancement*

| | | | |
|---|---|---|---|
| Stage I (4) ; | Average survival | = 8 years : | 5-year cure (3) |
| Stage II (2) ; | ,, ,, | = 13 years, 6 months : | 5-year cure (2) |
| Stage III (22) ; | ,, ,, | = 3 years, 4 months : | 5-year cure (5) |

In Wainwright's (1927) review, the average 5-year survival was 19 per cent. Huggins and Taylor (1955) state that their criteria for a cure consist of freedom from disease 5 years after treatment and subsequently ; for their primary cases a 12 per cent cure rate was given. For their " favourable " cases however, the figure rises to 35 per cent. In other recorded series Somerville (1952) gives 27·4– 5-year survival and 14·3–10-year survival ; Treves and Holleb (1955) 41·9, but here the 5-year survival only refers to strictly " operable " cases, whereas when all stages are taken into consideration it falls to 29·1 per cent.

*Treatment*

The treatment policy at the Royal Marsden Hospital for cancer of the *female* breast includes strict criteria of operability. Only those cases in the categories T1, T2, NO1 are considered suitable for a radical mastectomy. In all other cases, a course of high-voltage therapy is given and the position reassessed carefully three months after the conclusion of treatment. If these strict criteria of operability are applied in the male, then in this series only 3 patients out of the 15 who were treated by radical mastectomy would have been considered suitable for this operation; the remaining 12 cases were all T3. On the other hand, axillary lymph node involvement does not present this problem, since 19 cases in the series were either NO or N1 and only 4 of those treated by radical mastectomy were classified as N2.

It is suggested, therefore, that as far as the primary tumour is concerned, the indications for radical mastectomy include T3 cases, providing a wide excision with skin-grafting is carried out. The axillary lymph nodes on the other hand, should be dealt with in the same way as in the female, that is to say, N2 cases should be treated by radiotherapy and the position carefully reviewed three months later.

In this series, simple mastectomy with radiotherapy gives results comparable with those obtained by radical mastectomy and is certainly indicated in all patients over the age of sixty-five. Radiotherapy only, should be reserved for those cases which are inoperable when first seen and remain so after treatment.

It has not been possible to draw any conclusions as to the results of orchidectomy, since this operation has generally proved unacceptable to all those patients who have been advised to undergo it. However, treatment with corticosteroids seems to give results comparable to those in the female using oestrogen therapy and is recommended as a method of inducing temporary regression.

### SUMMARY

An unselected series of 28 cases of cancer of the male breast, treated at the Royal Marsden Hospital from 1936 to 1963, has been reviewed. The average age in the series was 60 years. All cases were verified by histological examination and fully documented. The tumours were graded according to Bloom (1950); there were 5 cases in Grade I, 16 in Grade II and 5 in Grade III. A significant family history was noted in 5 cases. Previous injury of some severity was found in 6 cases. No relationship could be established between the administration of hormones and the onset of cancer of the breast, nor were any benign mammary swellings, or gynaecomastia noted in any of the cases. The clinical features are characterised by advanced local disease, quite out of proportion to the degree of involvement of the axillary lymph nodes.

The results of treatment depend more on the grade of the tumour than on the stage of advancement of the disease and are improved by confining radical surgery to selected cases. In this series a 5-year survival of 36 per cent and a 10-year survival of 21 per cent were obtained.

We would like to thank Dr. Gowing and Dr. Hamlin of the Department of Pathology, Mr. Gibbons, the Medical Artist, and Mr. Vince of the Photographic Department for their valuable help. We are indebted to the Medical Committee of the Royal Marsden Hospital for access to hospital records.

### REFERENCES

ABRAHAMSON, W. AND WARSHAWSKY, H.—(1948) *J. Urol.*, **59**, 76.
BLOOM, H. J. G.—(1950) *Brit. J. Cancer*, **4**, 259.
BRYAN, R. C.—(1914) *Surg. Gynec. Obstet.*, **18**, 545.
GILBERT, J. B.—(1933) *Ibid.*, **57**, 451.

GREEN, W. AND NELSON, J. H.—(1953) *Amer. J. Surg.*, **85**, 431.
HUGGINS, C. Jr. AND TAYLOR, G. W.—(1955) *Arch. Surg.*, **70**, 303.
LACASSAGNE, A.—(1932) *C. R. Acad. Sci., Paris*, **195**, 630.
LUNN, J. R.—(1896) *Trans. path. Soc. Lond.*, **48**, 247.
MCCLURE, J. A. AND HIGGINS, C. C.—(1951) *J. Amer. med. Ass.*, **146**, 7.
PATEY, D. H. AND SCARFF, R. W.—(1928) *Lancet*, i, 801.
SACHS, M. D.—(1941) *Radiology*, **37**, 458.
SOMERVILLE, P.—(1952) *Brit. J. Surg.*, 39, 156, 296.
TREVES, N. AND HOLLEB, A.I.—(1955) *Cancer*, **8**, 1239.
WAINWRIGHT, J. M.—(1927) *Arch. Surg.*, **14**, 836.
WILLIAMS, I. G.—(1942) *Lancet*, i, 701.